# Comparison of Bioinformatics Approaches for Fetal Microdeletions and Monogenic Variations Estimation in Non-invasive Prenatal Testing

**DOI:** 10.1055/s-0042-1743573

**Published:** 2022-02-25

**Authors:** Lizzy Teleboshe Paul, Mahmut Cerkez Ergoren

**Affiliations:** 1 Department of Medical Biology, Faculty of Medicine, Near East University, Nicosia, Cyprus; 2 Department of Medical Genetics, Faculty of Medicine, Near East University, Nicosia, Cyprus; 3 DESAM Research Institute, Near East University, Nicosia, Cyprus

**Keywords:** prenatal testing, fetal DNA fraction, noninvasive prenatal testing

## Abstract

Prenatal testing provides crucial information about the health status of fetuses as well as recommending better treatment. For the past decades, prenatal testing using chorionic villus sampling and amniocentesis were the two majorly used forms of invasive prenatal diagnostic approaches. However, to facilitate prenatal testing without causing any danger to the fetus, the noninvasive prenatal diagnostic method, which uses circulating cell-free deoxyribonucleic acid (DNA), has become a suitable method of prenatal diagnosis. This review discusses the recent bioinformatics approaches used for analyzing fetal DNA concentration.

## Introduction

Prenatal testing is an eminent form of human prenatal care that is categorized into two types: (1) the prenatal diagnosis and (2) prenatal screening. Both the two forms of human prenatal testing are mainly used to assess pregnancy complications that can create physiological or morphological damage to the developing fetus or embryo at the early stage of gestation. Theoretically, prenatal testing is commonly used to screen for chromosomal anomalies or genetic mutations, as well as neural tube defects that can potentially lead to a series of various genetic aberrations and other birth deformities such as spina bifida, anemia, Down syndrome, cystic fibrosis, thalassemia, and muscular dystrophy in viable fetuses. 1

Prenatal screening tests focus on detecting anomalies that may occur during the fetal development at an affordable price. While the most well-known traditional forms of pregnancy screening are blood testing and ultrasound, known as double, triple, and quad screening, however non-invasive prenatal screening ideally provides more detailed information about already identified pregnancy-related issues. One of the conventional forms of prenatal diagnostic technique is the chorionic villus sampling (CVS), which utilizes placental tissue at 10 to 13 weeks of gestation to analyze chromosomal aberration with the aid of other technological platforms such as fluorescent in situ hybridization or polymerase chain reaction (PCR). 2 Another diagnostic technique is the amniocentesis method of testing, which utilizes amniotic fluids containing tissue to evaluate genetic anomalies. 3 CVS and amniocentesis are well known invasive prenatal diagnosis approaches that were previously associated with miscarriages risk. This prompted the development of the noninvasive prenatal diagnosis in recent years. 5

The newly developed non-invasive prenatal testing (NIPT) approach is a highly effective technique for analyzing fetal DNA through the use of cell-free fetal DNA (cffDNA) materials present in maternal blood. The cell-free DNA is of maternal DNA molecules circulating in the hematopoietic system of the expectant or pregnant women. 6 Additionally, the fetal DNA is derived from cytotrophoblastic cell apoptosis at the fetal stage of development. 7 Besides, since the discovery of cell-free DNA, different prospective forms of noninvasive methods of testing including highly efficient separation and massively parallel technologies such as next-generation sequencing and whole genome approaches for the detection of fetal anomalies have been developed. 8 Unlike the previous invasive prenatal diagnostic approaches (chorionic villus and amniocentesis), the newly invented NIPT method of testing allows fetal tissue examination without any risk to the fetus. 1 Also, for cfDNA-based NIPT method of testing, the quantity of fetal DNA sample present in the total cfDNA molecule obtained from pregnant women is expressed as the fetal fraction of the DNA, which is preeminent for the comprehensive performance of the NIPT analysis. 9 Additionally, during NIPT analysis for aneuploidy, the extracted fetal DNA fraction from maternal plasma is inversely proportional to the degree or magnitude of chromosomal anomalies present in plasma of the expectant. 9 Noninvasive tests can also be used to identify a fetus with monogenic diseases, 10 a group of genetic mutations in a single gene that can be inherited as autosomal recessive, autosomal dominant, or x-linked recessive, while in some minor cases, multiple mutations within a gene can cause the disease. Furthermore, these genetic changes can occur in a spontaneous manner, even so within or between families without previous history of the disease. The cff-DNA based NIPT for monogenic diseases is more challenging compared to chromosomal anomalies such as aneuploidies. This relies the difficulties on detecting single nucleotide changes from low amount of cfDNA. However, recently, a more convenient method called the relative haplotype dosage analysis (RHDO), a technique that generally uses detailed information of parental haplotypes located at the flanking regions of the gene of interest, has been developed. 9 The purpose of this review article is to compare the different bioinformatic approaches for fetal microdeletions and monogenic variation estimation in NIPT.

## Bioinformatics Approaches for Microdeletions and Monogenic Variation in NIPT

### Fetal DNA Detection Using Y-chromosome Approach

The method utilizes PCR assays to determine the fraction of fetal DNA sequence on the human Y-chromosomes compared with those found on the autosome. 11 However, for high-throughput Y-chromosome-based sequencing approach, the total DNA sequence reads found on Y chromosome is usually translated to the total fetal DNA fraction. The major disadvantage of this method is that it can only be applicable to pregnant women carrying male fetuses. 12

### Fetal DNA Estimation Using Parental Genotype–Based Approach

This method uses the parental genotype–based approach to analyze sequence reads for fetal-specific alleles present in the maternal plasma. Summarily, all fetal genotypes are heterozygous at a single nucleotide polymorphism (SNP) loci, while both parents are homozygous with different genotypes, for example, C/C for maternal genotype and A/A for paternal genotype. Using this approach, the amount of fetal DNA fraction is estimated by computing the proportion of fetal-specific alleles (A) to the sum total of alleles in the obtained plasma samples. 13 Although, this technique is one of the most suitable approaches for estimating the fetal DNA fraction, whereas rising two major disadvantages due to the requirement of specification of parental genotypes. Firstly, only the maternal blood samples are mostly used for NIPT analysis. Second, the genotype of the birth father (biological father) might not be obtainable during the test. 14

### Detection Using High Depth Sequencing of Maternal Plasma DNA

This method was recently developed to compensate for the requirement of parental genotype–based approach. This method utilizes the fetalQuant technology to measure the amount of fetal DNA using massively parallel sequencing of maternal plasma DNA. 15 For this approach, a model of binomial mixture is used to match the allelic frequency counts observed by the use of four different types of genotype combination obtained from maternal plasma DNA; this includes the AAaa, ABaa, ABab, and AAab genotypic markers to estimate fetal DNA concentration within a sample. A disadvantage of this method is that it requires a high debt detection of ∼120x to determine the targeted fetal alleles of interest. 9

### Shallow Depth Sequencing Data of Maternal Plasma Using Maternal Genotype–Based Approach

This method is a more advanced version of the fetalQuant technology that was developed for shallow depth sequencing data obtained through maternal genotype–based technique. 16 The main concept of this approach is based on the hypothesis that any nonmaternal allele found at an SNP locus where the expectant is homozygous would likely indicate a fetal-specific DNA allele. For example, the microarray technology in this case is used to identify all the sites where the pregnant woman is homozygous for through genotyping of a small amount of the maternal blood cells. Therefore, any plasma DNA molecule that varies from the sites where the pregnant woman is homozygous for are thought to be derived from the father. The estimate between the amount of nonmaternal alleles and the ratio of the actual fetal DNA obtained through parental genotyping is calculated using the linear regression model and the independent validation datasets with *r* = 0.9950 and a *p* -value of <0.0001 (Pearson's correlation). 9 One of the major advantages of this approach is that, once a reverential model is attained, it can be easily applied to other datasets obtained using the same sequencing or genotyping platforms as long as the population is the same. On the contrary, the technique is not applicable to datasets obtained from different sequencing or genotyping platforms as error rates can vary widely as well as the degree of heterozygosity among different ethnic groups.

### Detection Using SeqFF DNA Sequencing Data Approach

In recent years, a more reliable technique for shallow depth maternal plasma DNA sequencing approach, called SeqFF, was developed. The method is less time-consuming and effortlessly used to evaluate the actual fetal DNA fraction from the routine datasets of NIPT procedures. In general, the method uses a single end of randomly sequenced maternal DNA plasma read counts of 50 kb of all the autosomal regions fitted into a high-dimensional regressive model. 17 The standardized 50-kb reads, which are used as predictor variables, are derived from other chromosomes except chromosomes 21, 13, 18, Y, and X. The model coefficient of this technique is determined through the use of a reduced rank regression and elastic net (Enet) models. 17 Peng et al (2017) reported a similar finding for Y-chromosome-based approach and the SeqFF-based approach after analyzing two independent cohorts ( *r* = 0.938 and 0.932, respectively, using Pearson's correlation). 9 One disadvantage of this approach is the need for high-dimensional model as it requires large amount of sample size for fetal DNA fraction estimation.

### Detection Using Cell-Free DNA Size based Approach

Several studies have reported that maternal- and fetal-derived DNA molecules present within plasma samples are not of the same in lengths, 8 9 18 stating that fetal DNA are likely shorter than maternal DNA in length. 8 18 Hence, higher concentration of fetal DNA molecules would likely increase the percentage of shorter fragments. Yu et al (2014) used paired end sequencing to develop a method to estimate fetal DNA molecules. They recorded average read counts of ∼100 to 150 bp and 163 to 169 bp with optimal performance. 19 Also, the authors used a linear regression model on 36 datasets to estimate the size ratio and the total amount of fetal DNA sample by quantifying the sequence reads obtained from Y chromosomes after which they used a derived model to translate the size ratio of the datasets in fetal DNA fractions. 19

### A Technique Using Methylation Marker to Estimate Fetal DNA Concentration

This approach is based on the DNA methylation epigenetic modification process where a methyl group is added to a cytosine base of the DNA sequence of mammalian organisms. DNA methylation of the cytosine occurs at ∼70% and it is believed that most organs can be identified based on their differential methylation status. 20 Therefore, due to the speculation of differential methylation states, a placental methylation–specific markers-based technique for fetal DNA estimation was developed. 9 Nygren et al (2010) used five different methylation regions to compare placental tissues alongside a CGP island microarrays and methyl-cytosine immunoprecipitation to mine maternal plasma buffy coat. 21 After this, a quantitative assay was used to calculate the concentration of fetal DNA fraction in the maternal plasma sample. Additionally, Lun et al (2013) used a massively parallel bisulfite sequencing to determine the fetal DNA fraction through the use of fetal-derived DNA material within variable methylated regions. 22 23

### Fetal DNA Estimation Using Nucleosome Track Method

In recent years, the evaluation of the nucleosomal origin of plasma DNA has progressively been recognized as a suitable method for fetal DNA estimation and has been discussed in two studies where high-resolution size profiling of maternal plasma DNA was used to investigate cell-free DNA. 9 In a study by Straver et al (2016) in which they analyzed maternal plasma DNA sample from 298 cases based on the nucleosome tracker hypothesis, they found a correlation between fetal DNA concentration and the prevalence of reads from => 73 bp (upstream and downstream) inferential center. 24

## Conclusion

To date, there have been various technological approaches and different bioinformatics algorithms developed for assessing circulating DNA (cf DNA). More importantly, with the availability of next-generation sequencing technologies that provide a fast, easy, efficient, precise, and low-cost method of assessing fetal DNA fraction, NIPT has evolved as a prenatal testing method in revolutionary medicine. This article compared not all, but few important approaches for fetal DNA estimation. However, additional studies on the above-mentioned approaches will be required to provide exceeding knowledge regarding the concepts of fetal DNA estimation. It will also help elucidate the dominant factor of fetal DNA fraction in the pathogenesis of different diseases.
